# Mitochondrial Dysfunction in Cardiovascular Diseases: Potential Targets for Treatment

**DOI:** 10.3389/fcell.2022.841523

**Published:** 2022-05-13

**Authors:** Jiaqi Yang, Qianyun Guo, Xunxun Feng, Yang Liu, Yujie Zhou

**Affiliations:** Beijing Key Laboratory of Precision Medicine of Coronary Atherosclerotic Disease, Department of Cardiology, Beijing Institute of Heart Lung and Blood Vessel Disease, Clinical Center for Coronary Heart Disease, Beijing Anzhen Hospital, Capital Medical University, Beijing, China

**Keywords:** cardiovascular diseases, mitochondrial dysfunction, reactive oxygen species, mitophagy, mitochondrial therapy

## Abstract

Cardiovascular diseases (CVDs) are serious public health issues and are responsible for nearly one-third of global deaths. Mitochondrial dysfunction is accountable for the development of most CVDs. Mitochondria produce adenosine triphosphate through oxidative phosphorylation and inevitably generate reactive oxygen species (ROS). Excessive ROS causes mitochondrial dysfunction and cell death. Mitochondria can protect against these damages *via* the regulation of mitochondrial homeostasis. In recent years, mitochondria-targeted therapy for CVDs has attracted increasing attention. Various studies have confirmed that clinical drugs (β-blockers, angiotensin-converting enzyme inhibitors/angiotensin receptor-II blockers) against CVDs have mitochondrial protective functions. An increasing number of cardiac mitochondrial targets have shown their cardioprotective effects in experimental and clinical studies. Here, we briefly introduce the mechanisms of mitochondrial dysfunction and summarize the progression of mitochondrial targets against CVDs, which may provide ideas for experimental studies and clinical trials.

## 1 Mitochondrial Pathophysiology and Reactive Oxygen Species (ROS) Generation in the Cardiovascular System

Mitochondria are semiautonomous organelles located in the cytoplasm. Unlike other organelles, mitochondria have their own genomes (mtDNAs), double-stranded circular DNAs, and are coated by a double-layered membrane (Davis and Williams, 2012; van der Bliek et al., 2017). Mitochondria are responsible for the production of adenosine triphosphate (ATP) and the regulation of nutritional metabolism, calcium homeostasis, and cellular viability in several organs (Kim et al., 2008). Due to the huge demand for ATP in the heart, mitochondria are highly abundant in cardiac cells, especially in cardiomyocytes (CMs), occupying nearly 20–40% volume in adult CMs (Schaper et al., 1985). Approximately 6 kg of ATP is produced by cardiac mitochondria every day (Siasos et al., 2018). Thus, mitochondria play essential roles in the cardiovascular system.

**FIGURE 1:**
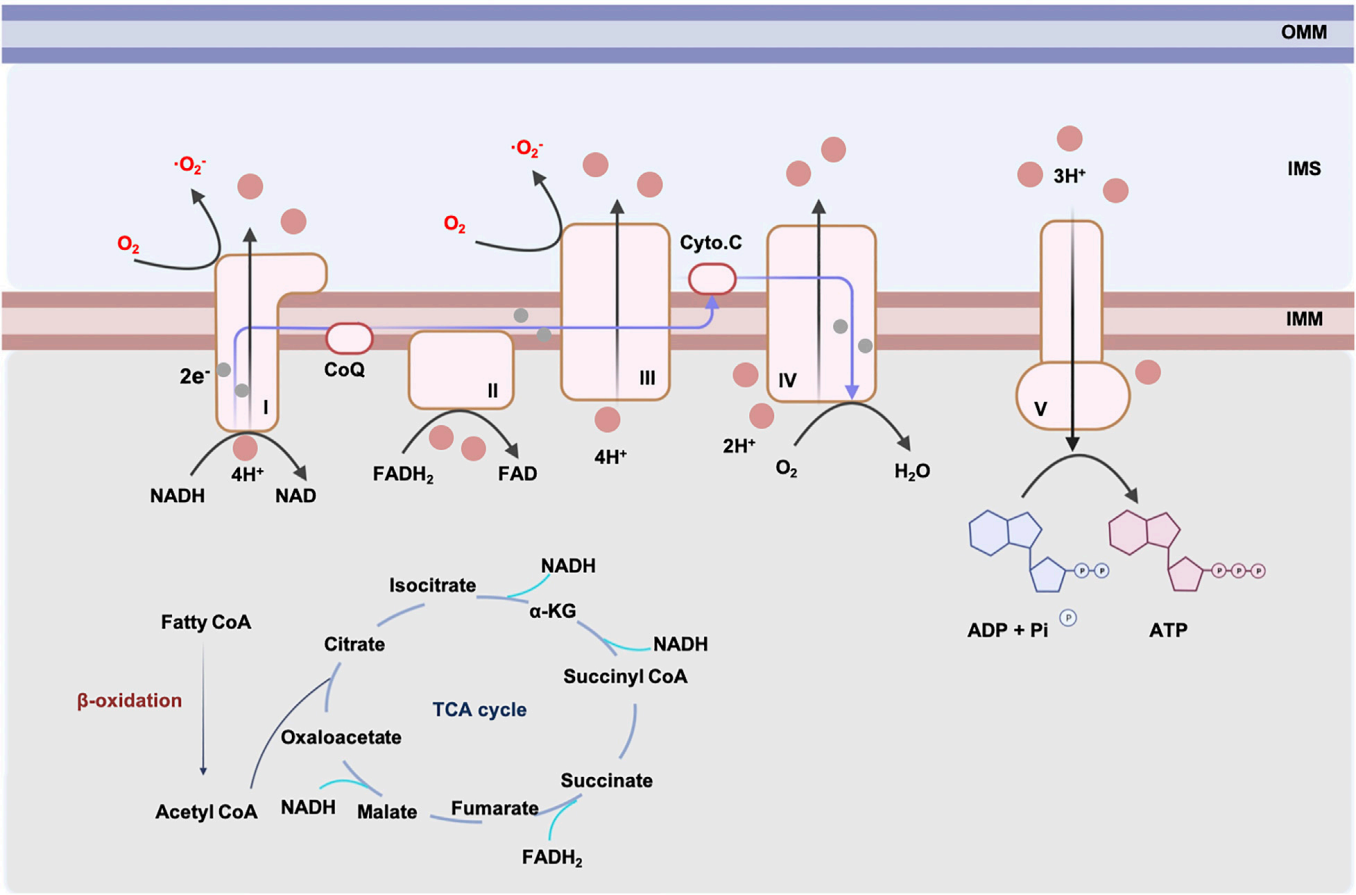
ATP generation and ROS production in cardiac mitochondrion. The major resource of cardiac mitochondria comes from β-oxidation. Generation of NADH and FADH_2_ from β-oxidation and TCA cycle are used by respiratory chain complexes. Respiratory chain complexes are located in IMM. Electrons generated from complex I, II pass through the respiratory chain and alter the concentration gradient of H^+^. Complex V produces ATP driven by H^+^ flow. During the generation of ATP, complex I and III produce O_2_^−^. Excessive ROS leads to mitochondrial dysfunction. Abbreviations: IMM, inner mitochondrial membrane, OMM, outer mitochondrial membrane, IMS, mitochondrial intermembrane space, ROS, reactive oxygen species, NADH, reduced nicotinamide adenine dinucleotide, FADH2, reduced flavin adenine dinucleotide, CoQ, coenzyme Q, Cyto. C, cytochrome C, ADP, adenosine diphosphate, ATP, adenosine-triphosphate triphosphate, TCA, tricarboxylic acid cycle, CoA, coenzyme A, α-KG, α-ketoglutarate.

The main source of energy obtained by oxidative phosphorylation (OXPHOS) comes from the oxidation of fatty acids in the adult heart (Stanley, 2005). ATP is synthesized in adult hearts following the steps below: fatty acyl-coenzyme A (CoAs) are synthesized with the help of acyl CoA syntheses. To enter the cardiac mitochondria, fatty acyl CoAs are converted to acylcarnitines by carnitine palmitoyl transferase 1 (CPT1) and are transferred to the inner membrane of mitochondria (IMM) where they are liberated to fatty acyl CoAs and initiate β-oxidation. Acetyl CoA, a product of β-oxidation, enters the Kerbs cycle and generates reduced nicotinamide adenine dinucleotide (NADH) and reduced flavin adenine dinucleotide (FADH_2_). NADH and FADH_2_ generated from the Kerbs cycle, and from β-oxidation, transfer electrons through the electron transport chain (ETC), which comprises four complexes on the IMM. The efflux of protons accompanied by the electronic flow on ETC activates ATP-synthase and produces ATP (shown in Figure 1) (Chen and Butow, 2005).

However, the production of ATP through the respiratory chain is accompanied by an inevitable generation of ROS, especially superoxide anion (·O_2_^−^). It has been reported that nearly 1, 2% of electrons is related to superoxide production during ATP synthesis (Boveris et al., 1976). Complex I and III are the main sites of ROS production in mitochondria (Cadenas, 2018). Complex I is an L-shaped component made up of a hydrophilic arm and a hydrophobic arm, in which the semi-/reduced flavin mononucleotide (FMN) and N1a/N1b iron-sulfur clusters are the main sites for superoxide production (Efremov and Sazanov, 2011). Complex III generates superoxide mostly by ubisemiquinone formed at the Q_o_ site, where ubisemiquinone transfers electrons to oxygen (Iwata et al., 1998). ROS generated from mitochondrial complex I and III is the main mechanism of ischemia/reperfusion (I/R) injury in the heart. Kang et al. reported that complex I is responsible for the generation of O_2_^−^ in post-ischemic CMs, while damage to the N1a cluster causes FMN-produced superoxide (Kang et al., 2018). Meanwhile, Chen et al. found an increasing generation of antimycin A-enhanced superoxide in complex III *ex vivo* (Chen et al., 2008). Normal amounts of O_2_^−^ can be converted to more stable hydrogen peroxide by superoxide dismutases (SODs) (Suzuki et al., 2013). However, excessive O_2_^−^ can damage mtDNA, lipids, and proteins, leading to mitochondrial dysfunction, oxidative stress, and cell death (Haworth and Hunter, 1979; Peoples et al., 2019). In cardiovascular diseases (CVDs), it has been reported that excessive ROS and mitochondrial dysfunction are related to numerous cardiac diseases, such as atherosclerosis (AS), I/R injury, cardiac hypertrophy, heart failure (HF), and degenerative aortic valve disease (Peoples et al., 2019).

**FIGURE 2:**
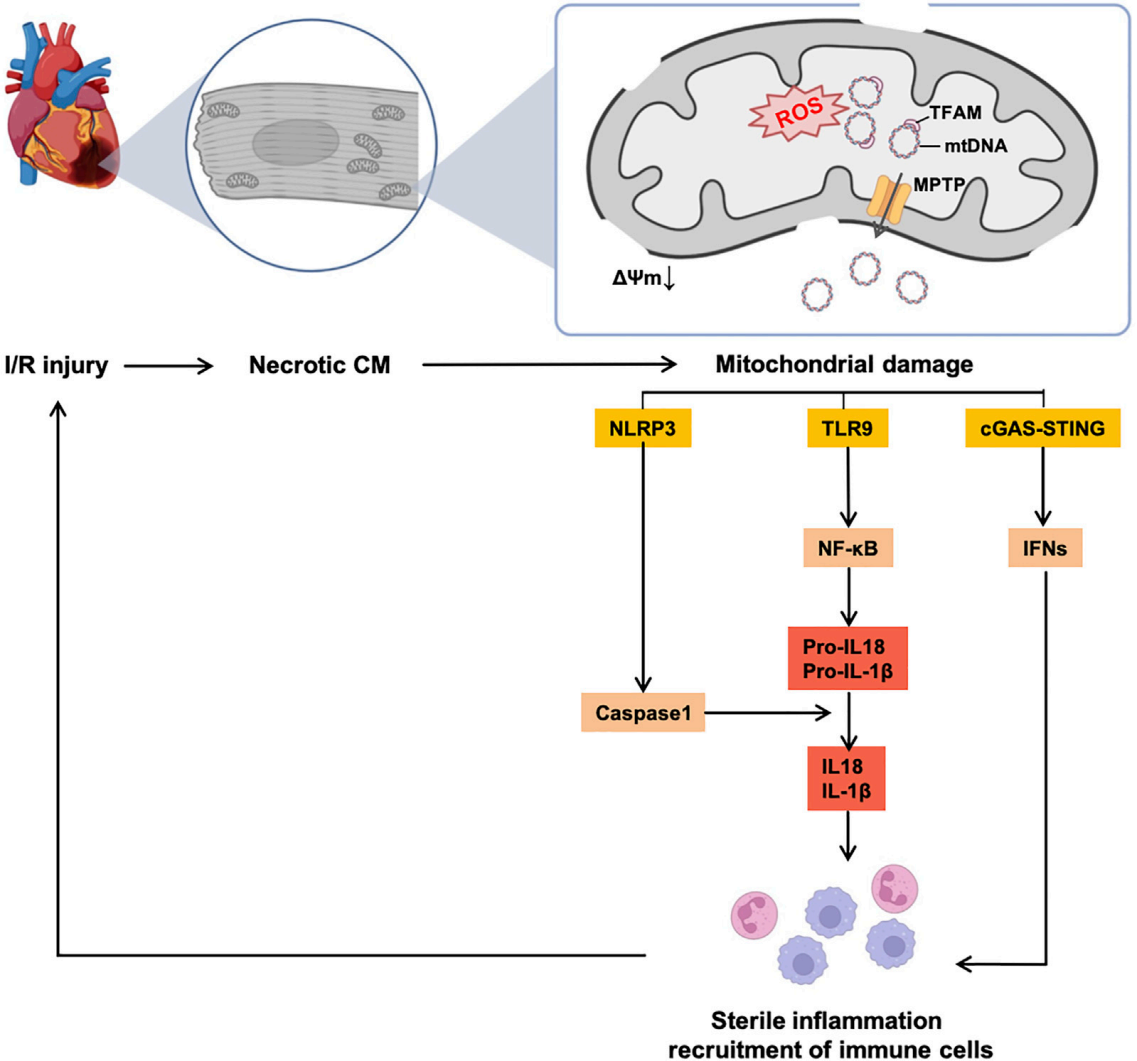
Vicious circle of mtDNA-induced sterile inflammation and mitochondrial dysfunction in the I/R heart. Accumulation of mutant mtDNAs caused by excessive ROS release from MPTP, thereby inducing consecutive sterile inflammatory responses and recruitment of immune cells, which continuously aggregate I/R injury and CM necrosis. Abbreviations: I/R, ischemia/reperfusion, CM, cardiomyocyte, NLRP3, nod-like receptor pyrin domain containing 3, TLR9, toll-like receptor 9, cGAS, cyclic GMP–AMP synthetase, STING, stimulator of interferon gene, NF-κB, nuclear factor kappa B, IFN, interferon, IL, interleukin.

The classical perception is that ROS is the major driver of the mutations of mtDNA, while others are replicated errors of mtDNA, and present a less efficient mtDNA repair system than nuclear DNA. mtDNA, which is closed to ROS and ETC, causes the degradation of nucleic acid chemical components. The most common induction of mutations is *via* point mutation of purines and pyrimidines (Lee and Wei, 2007). An accumulation of multiple mutations leads to the exhaustion of mtDNA-encoded proteins and less efficient ATP production. It is also recognized that accumulated mtDNA damage can also increase ROS generation, resulting in a vicious circle (Płoszaj et al., 2010). Additionally, mitochondria trigger and sustain the chronic-sterile inflammation and immunity in the heart, which mostly occur after I/R damage (shown in Figure 2), and are related to the development of HF (Nakayama and Otsu, 2018). This phenomenon is induced by activated pattern recognition receptors (PRRs, e.g., toll-like receptor 9 [TLR9] and nod-like receptor pyrin domain containing 3 [NLRP3]) sensing damage-associated molecular patterns (DAMPs) (Rodrigues et al., 2015). As for the characteristics of mtDNA (close to the respiratory chain, sensitive to ROS and a relatively low level of methylation), the accumulation of mtDNA mutations represents the most well-known DAMP, which activates PRRs, recruits inflammatory cytokines, and activates the response of immune cells in the heart.

Mechanisms of mtDNA release from mitochondria remain controversial. The classical theory believes that the mtDNA release is mediated by the opening of the mitochondrial permeability transition pore (MPTP) (Patrushev et al., 2004). The MPTP is an unspecific pore in the mitochondria initially found by Haworth et al. (Haworth and Hunter, 1979). MPTP is strongly related to acute I/R injury and is open under conditions of oxidative stress, overloaded [Ca^2+^]_mito_ and altered phosphate level during ischemia (Crompton et al., 1987). Although the opening of the MPTP is not ROS-dependent, excessive ROS synergizes the opening of the MPTP with other factors (mentioned above), thereby leading to further mitochondrial injury (Haworth and Hunter, 1979). In addition, evidence has shown that inhibiting the MPTP is an essential mechanism of cardioprotective ischemic pre-/post-conditioning (*iPre* and *iPost*) in the heart (reviewed by Penna et al.) (Hausenloy et al., 2009). Opening of the MPTP causes the swelling of the mitochondrial matrix and damage to the outer mitochondrial membrane (OMM), therefore leading to the release of pro-apoptotic factors. Especially, the opening of the MPTP is the major way of releasing mtDNAs from mitochondria and induces sterile inflammation. Therefore, the MPTP is recognized as a promising target against mitochondrial dysfunction in the heart, and several studies from animal models and clinical trials have investigated this target (details are in Section 3.4).

It is also reported that mtDNA release is a voltage-dependent anion channel (VDAC) oligomers-regulated process. Mitochondrial outer membrane permeabilization (MOMP) is required for the release of the mtDNA. Kim et al. found that VDAC can oligomerize under the stimulation of oxidative stress, and induce the MOMP as well as the liberation of mtDNA (Kim et al., 2019). Riley et al. reported that the release of the mtDNA is also related to the mitochondrial inner membrane permeabilization (MIMP). A Bax/BAK (pro-apoptotic member B-cell lymphoma 2, Bcl-2 family)-induced expansion of OMM pores following behind MOMP causes extrusion of IMM from mitochondrion to cytoplasm and the occurrence of MIMP-mediated mtDNA release (Riley et al., 2018). Besides, mitochondrial-derived vesicles (MDVs) have been described as an additional regulator of mitochondrial homeostasis (the major mechanisms are mentioned in Section 2), which establish crosstalk between mitochondria and lysosomes. Dysfunctional mitochondria could lead to leakages of MDVs including their components, which trigger PRRs and activate inflammation. However, the hypothesis of MDVs-dependent mtDNA release remains under investigation (Picca et al., 2019; Picca et al., 2021).

In the next step, mutant mtDNA can be released into intracellular and extracellular spaces. For intracellular liberation, damaged mtDNAs released from disruptive OMM to the cytoplasm, which activates intracellular PRRs and initiates the non-infectious inflammation *via* interleukin 10 (IL-10) and IL-1β. mtDNA can also induce the activation of cyclic GMP–AMP synthetase (cGAS)/stimulator of interferon gene (STING)/type I interferon (IFN) pathways without inflammatory cytokines activation, especially in the I/R injured heart (White et al., 2014). Several pieces of evidence support the statement that mtDNA can also release out of cells, activating inflammation and stimulating an immune response. In necrotic cells, mtDNA leaks through the disruptive plasma membrane into extracellular space. There are some other mechanisms for the liberation of mtDNA from non-necrotic cells. Guescini et al. detected the existence of cell-free mtDNA in exosomes released by glioblastoma cells and astrocytes, which implies exosomes proceed with the extracellular release of mtDNA (Guescini et al., 2010). Lood et al. reported the release of neutrophils extracellular traps (NETs) is also accompanied by the liberation of mtDNA (Lood et al., 2016). Extracellular leakages of mtDNAs can be recognized by TLR9 through endocytosis or directly captured by TLR9 on the plasma membrane of some cells (e.g., resting B cells and peripheral blood monocytes) and activate inflammation (Eaton-Bassiri et al., 2004; Dasari et al., 2005).

It is not surprising to see that accumulated mtDNA damages and mtDNA-induced chronic inflammation are correlated to AS, I/R injury, hypertension and HF due to the high consumption of oxygen and ATP in the heart (Poznyak et al., 2020). Among them, AS is strongly related to mtDNA mutations. Primary AS (AS that cannot be explained by classical risk factors, such as diabetes, hyperlipidemia, and smoking) has been described as a result of mitochondrial disorder. A study analyzed twelve lipofibrous plaques of aorta intima compared to intact area, and reported four mtDNA mutations, including m.1555A > G, m.3256C > T, m.12315G > A and m.15059G > A, are relevant to atherosclerotic plaques (Sobenin et al., 2013). An analysis enrolled 65 blood samples and 23 atherosclerotic plaques from coronary artery disease (CAD) patients showed a higher deletion of 4977bp (26.2% *vs* 4.5%) as well as higher heteroplasmy rates (18–46%) than the control group. Interestingly, these deletions are independent of classical risk factors of AS and not inferred by baseline characteristics of patients (Sobenin et al., 2013). Furthermore, Sazonovo et al. showed the data from 225 myocardial infarction (MI) patients, that a mutation of m.5178C > A is related to the occurrence of MI. On the opposite, the other two variants of m.14846G > A and m.12315G > A are observed in healthy controls (Sazonova et al., 2018). A syndrome of Mitochondrial Encephalomyopathy, Lactic Acidosis, and at least one Stroke-like episode (MELAS) is one of the representative mitochondrial diseases carrying a mutant of m.3243A > G in mitochondrial tRNA leucine 1 (Manwaring et al., 2007). Numerous studies found patients with MELAS are also accompanied by dysfunctional endothelia, atherosclerotic carotid, and cerebral artery without potential risk factors of AF. These studies also provide indirect proof of the development of mutant mtDNA-related AS (Pek et al., 2019; Finsterer, 2020). Besides, mtDNA damages also play an essential role in the development of AS accelerated by traditional risk factors mentioned above. A review from Sobenin et al. well summarized the variants of mtDNA relevant to smoking, MELAS, and diabetes mellitus, which are the promoters of the development of AS (Sobenin et al., 2015).

On the other hand, chronic inflammatory and immune responses induced by mtDNA-related DAMPs are also involved in AS (Sobenin et al., 2015). mtDNA mutations affect many cell types in AS. In endothelial cells (ECs), mtDNA activates TLR9 and induces the production of interleukin-1β (IL-1β) through the NF-κB pathway. Moreover, the damage associated with ECs recruits inflammatory cells and alters vascular wall permeability, which initiates the development of AS (Jin et al., 2019). The release of mtDNA induces the activation of NF-κB in macrophages and is related to the synthesis of pro-inflammatory cytokines, uptake of oxidized low-density lipoprotein (oxLDL), and formation of foam cells (Tedgui and Mallat, 2006). Dysfunction of mitochondrial dynamics induced by damaged mtDNA in smooth muscle cells (SMCs) results in their energetic decline and apoptosis in plaque, which alters the stability of plaque in AS (Yu et al., 2017; Docherty et al., 2018). Besides, the release of mutant mtDNA from damaged mitochondria to the cytoplasm is a sign of the early phase of AS. Yu et al. detected mtDNA in both circulating and vascular cells in human atherosclerotic plaques even before other signs of AS occur (Yu et al., 2013). Together all, mtDNA mutations and mtDNA-induced sterile inflammations are essential targets/aspects for the detection and treatment of AS.

## 2 Major Pathways Against Mitochondrial Dysfunction

**FIGURE 3:**
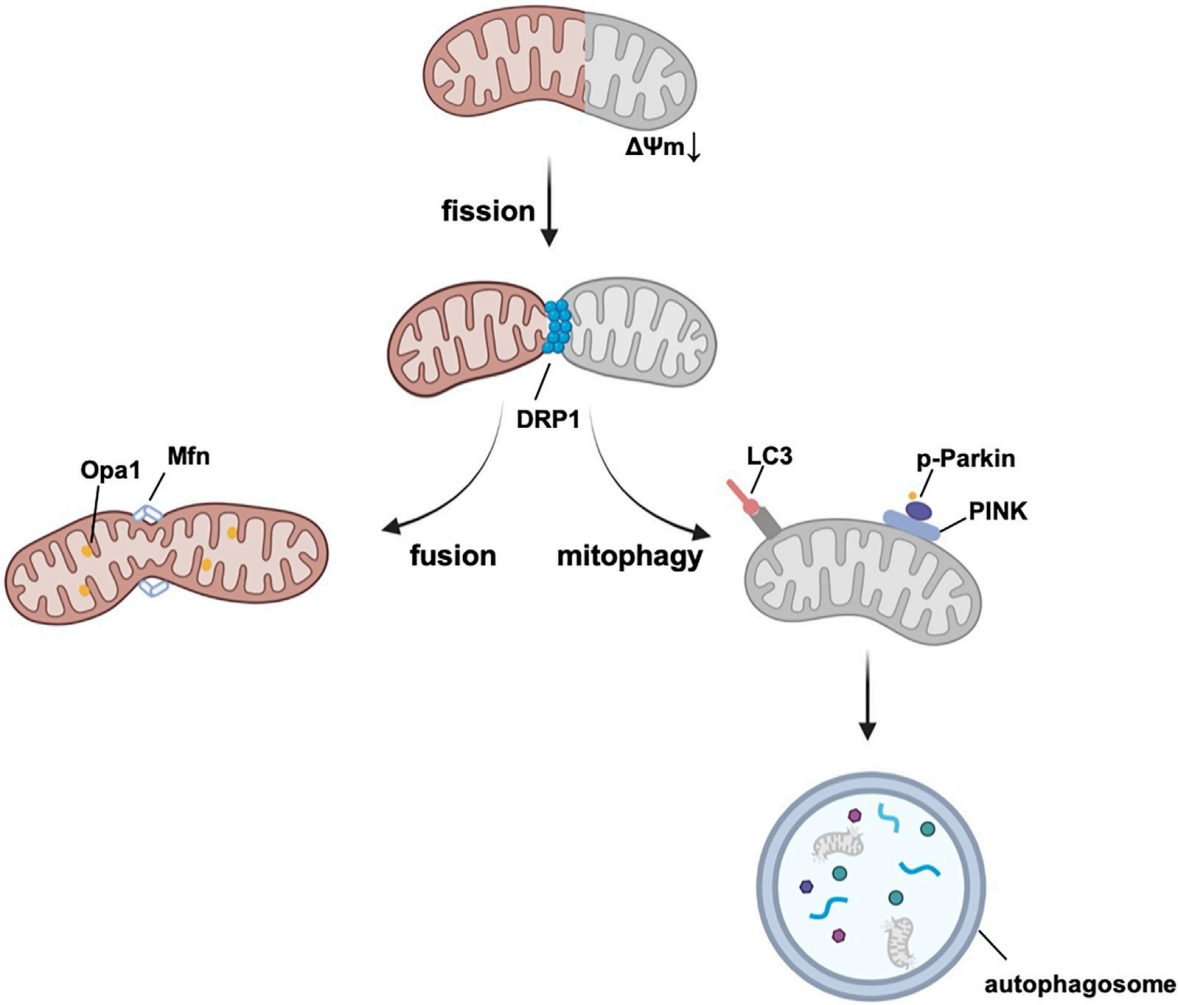
Mitochondrial quality control system. Damaged mitochondrial fragment splits from healthy mitochondrion by fission and then degrades by mitophagy. Mitochondrial fusion assembles healthy mitochondria and maintains the normal function of mitochondria. Abbreviations: Opa1, optic atrophy 1, Mfn, mitofusin, DRP1, Dynamin-related protein 1, LC3, microtubule-associated protein 1A/1B-light chain 3, PINK, PTEN-induced kinase 1, Δψm, mitochondrial membrane potential.

To avoid the accumulation of ROS and mitochondrial dysfunction, mitochondria sustain their homeostasis by mitochondrial biogenesis, mitochondrial dynamics, and mitophagy (summarized in Figure 3), all of which are potential targets for treatment.

Mitochondrial biogenesis increases the number of healthy mitochondria in the heart, improves the replication and repair of mtDNA, and induces the synthesis of mitochondrial enzymes and proteins (Popov, 2020). It has been reported that mitochondrial transcription factor A (TFAM) and peroxisome proliferator-activated receptor-γ coactivator-1α (PGC-1α) are central regulators of mitochondrial biogenesis (Jornayvaz and Shulman, 2010). PGC-1α activates TFAM, which is essential for the transcription, replication, and stability of mtDNA (Yokokawa et al., 2018). PGC-1α can also increase the expression of nuclear respiratory factor (NRF)-1 and NRF-2, thereby promoting the production of vital mitochondrial enzymes (Reznick et al., 2007). There are several pathways contributing to mitochondrial biogenesis. Jager et al. reported that adenosine 5‘-monophosphate-activated protein kinase (AMPK) is critical for mitochondrial biogenesis by regulating the phosphorylation of Thr177 and Ser538 (two sites of PGC1α) (Jäger et al., 2007). Sirtuins (SIRT) are a series of deacetylases and deacetylases that control energy metabolism. There is evidence that SIRT1 promotes the uptake of glucose and mitochondrial biogenesis by activating PGC-1α (Guarente, 2007). Wang et al. reported that SIRT1 can prevent heart aging by mediating deacetylation of PGC-1α and activation of mitochondrial biogenesis (Wang et al., 2020a). To summarize, morphological and quantitative changes in mitochondria have been observed in the heart, liver, and skeletal muscle in pathological conditions, and the regulation of mitochondrial biogenesis is a potential target to treat mitochondrial dysfunction.

Mitochondrial dynamics, represented by the balance of mitochondrial fission and fusion, are under the regulation of dynamin-related guanosine triphosphatase (GTPase) proteins (Hoppins et al., 2007). Dynamin-related protein 1 (DRP1) is the main protein of fission, which interacts with the receptors located in OMM, such as mitochondria fission factor (MFF), fission protein-1 (Fis1), and mitochondrial dynamics proteins 49 and 51 (MiD49/51) (Long et al., 2013; Richter et al., 2014; Kornfeld et al., 2018). Besides, MiD49/51 and MFF have also been reported to modulate GTPase-derived DRP1 constrictive activity (MiD49/51 inhibits GTPase, whereas, MFF promotes it) (Liu and Chan, 2015; Osellame et al., 2016). During the fission phase, dysfunctional fragments of mitochondria can be cut into small, spherical pieces and then degraded by mitophagy. Excessive mitochondrial fission is responsible for increasing the infarct area in I/R heart injury, and it induces both excessive mitophagy and cell death (Bi et al., 2019). Drugs such as metformin can block DRP1 and maintain the integrity of mitochondria according to the studies using diabetic animal models *in vivo* (Li et al., 2016; Wang et al., 2019). Recently, metformin is also demonstrated to protect against AS by reducing cardiac mitochondrial fission (it is most well recognized by its mechanism in the regulation of mitochondrial complex I, mainly through the AMPK pathway, discussed in 3.3) (Wang et al., 2017). Mitochondrial fusion is first mediated by mitofusin 1 (Mfn1) and Mfn2 in the OMM, and then by optic atrophy 1 (Opa1) in the IMM (Koshiba et al., 2004). Deletion of Mfn1 and Mfn2 in the embryo causes death *in utero*, indicating that Mfn1 and Mfn2 are strongly contributed to heart development (Chen et al., 2003; Chen et al., 2011). There is evidence from murine hearts that the ablation of Mfn1 and Mfn2 is related to the fragmentation and malfunction of mitochondria and induction of lethal cardiomyopathy (Papanicolaou et al., 2012). A decreasing level of Opa1 is found in post-MI hearts of both rats and humans and correlates with the downregulation of mtDNA and antioxidant genes (Chen et al., 2009). Mitochondrial fusion is a beneficial process in which mitochondria with tiny defects of proteins and DNA are complemented by functional parts of other mitochondria, and are merged into elongated, tubular, interconnected mitochondrial networks (Campello and Scorrano, 2010).

Mitophagy is a form of autophagy that selectively targets the degradation of dysfunctional mitochondria and serves as a control system of mitochondria, and autophagosomes are the main effectors during mitophagy (Lemasters, 2005). Together with mitochondrial biogenesis and dynamics, mitophagy sustains the homeostasis of mitochondria. At the cellular level, mitophagy shows effects on cell death of CMs (Shires and Gustafsson, 2015; Torrealba et al., 2017), and regulates the differentiation of cardiac fibroblasts (CFs), the main responding cell population in post-MI hearts, thereby controlling cardiac remodeling (Urban et al., 2015). Normal levels of mitophagy protect against I/R damage. Several studies have investigated that repeated and short *iPre* promotes the adaptation of cardiac mitochondria to I/R injury *via* mitophagy (Zhang et al., 2016; Zhang et al., 2017; Xiao et al., 2020). In addition, mitophagy contributes to the remission of other CVDs, such as hypertension, cardiac remodeling after MI and diabetic cardiomyopathy (more details are provided in the review by Morales et al.) (Morales et al., 2020). Several important pathways are involved in different phases of mitophagy. Mitophagy usually starts with mitochondrial imperfection and a reduction in mitochondrial membrane potential (Δψm). Regularly, PTEN-induced kinase 1 (PINK1) recognizes damaged mitochondria and accumulates in the OMM, thereby leading to the phosphorylation of Parkin E3 ligase. Parkin helps to ubiquitinate proteins of the OMM (Kondapalli et al., 2012). Mitochondrial cargo receptors (MCRs) guide these proteins to bind with microtubule-associated protein 1A/1B-light chain 3 (LC3), which can be recognized by autophagosomes and finally digested (Lazarou et al., 2015). Interestingly, DRP1 can also help selectively recognize damaged mitochondria from healthy population by mitochondrial fission. PINK sometimes cannot recognize the damaged part of mitochondrion easily due to the interconnected rapid balance of electrochemical gradients. However, transient reduction of Δψm can be a negative selective pressure for DRP1-induced fission. Healthy part of the mitochondrion will enter the fission-fusion cycle. Whereas, dysfunctional sectors which cannot recover back to normal Δψm can be negatively selected by mitophagy. Additionally, DRP1, together with Δψm, can also induce a positive feedback loop (reduction of Δψm recruits DRP1, recruited DRP1 ulteriorly reduces Δψm), which is helpful to selective mitophagy. (Cho et al., 2019). Other MCRs can directly stimulate mitophagy in ubiquitination-independent pathways. Cardiolipin is mostly synthesized on the IMM (Ren et al., 2014) and has effects on the respiratory chain and mitochondrial quality control system (mitochondrial dynamics and mitophagy) (Dudek, 2017; Paradies et al., 2019). Damage signals of mitochondria cause the translocation of cardiolipin to the OMM, which combines with LC3 and contributes to the recognition of dysfunctional (part of) mitochondria in the heart (Chu et al., 2013; Dudek et al., 2019). Furthermore, B-cell lymphoma-2 (BCL2)-interacting protein 3 (BNIP3) and FUN14 domain containing 1 (FUNDC1) have been reported to activate mitophagy *via* a PINK1-independent pathway and help to protect against oxidative injury and I/R in the heart (Liu et al., 2012; Zhang et al., 2016).

Based on the mechanisms of mitochondrial injury/repair mentioned above, we summarize mito-targeted modulators against mitochondrial dysfunction in CVDs.

## 3 Potential Targets Against Mitochondrial Dysfunction

### 3.1 Antioxidants Against ROS

Triphenylphosphonium (TPP^+^) is a lipophilic cation, which accumulates in mitochondria due to the Δψm. Drugs conjugated with TPP can concentrate up to 500-fold more in mitochondria than in the cytoplasm (Burns et al., 1995; Smith et al., 1999). Most of the antioxidants used for the treatment of mitochondrial dysfunction are TPP^+^-binding compounds, and MitoQ and SkQ are the representative TPP^+^-binding drugs.

MitoQ is co-enzyme Q10 (CoQ10) attached to TPP^+^ in C10 hydrophobic domain. CoQ10 has been well described as a global antioxidant and is beneficial to prevent hypertension and HF (Pepe et al., 2007). However, it is not clear how much CoQ10 can be uptake by mitochondria. MitoQ may solve the problem of non-targeted mitochondrial uptake, as it combines well with fatty acids and activates the transmembrane proton conduction in the IMM (Severin et al., 2010). Numerous studies have shown the antioxidant function of MitoQ and its mechanisms in HF. Junior et al. reported that MitoQ rescues the cardiac function of pressure-overloaded HF in a mouse model (Ribeiro Junior et al., 2018). Goh et al. described that MitoQ controls the expression levels of *Chast* and *Mhrt* and attenuates cardiac remodeling and prevents HF *ex vivo* (Goh et al., 2019). MitoQ also protects the function of Mfn2 by regulating redox-sensitive lncRNA-microRNA networks (e.g., Mfn2 targeted, Plscr4-miR-214) (Kim et al., 2020). Furthermore, MitoQ has been shown to alleviate I/R injury of mouse and rat hearts *ex vivo* (Adlam et al., 2005; Dare et al., 2015). Taken together, MitoQ displays impressive benefits in the treatment of aging diseases, I/R injury, HF, and obesity both *in vitro* and *in vivo* (Dietl and Maack, 2017; Braakhuis et al., 2018; Hu et al., 2018; Fink et al., 2021). As a potential target for treatment, numerous clinical studies (completed and ongoing) have attempted to investigate its effects on humans. Firstly, the safety and tolerability of MitoQ administration as a supplemental diet in young adults have been proven previously (Shill et al., 2016). Secondly, MitoQ shows mixed outcomes in different diseases but has the advantage of improving vascular function in aged adults. Rossman et al. reported their results of a clinical trial that administered MitoQ (up to 6 weeks), showing that MitoQ improved brachial artery flow-mediated dilation (FMD, a parameter of evaluating vascular function), and decreased aortic stiffness and plasma oxidized low-density lipoprotein (ox-LDL, a marker of oxidative stress); the underlying mechanism was the ability of MitoQ to ameliorate oxidative stress and promote endothelial function (Rossman et al., 2018). Furthermore, MitoQ treatment for up to 28 days in chronic hepatitis C showed decreased plasma alanine transaminase (ALT) aspartate aminotransferase (Gane et al., 2010). In contrast, another clinical study showed no improvement of MitoQ therapy on Parkinson’s disease (Snow et al., 2010). Currently, no completed clinical trial has focused on the effect of MitoQ on CVDs, especially in I/R injury in the heart. However, two clinical studies focused on cardiac function are ongoing (NCT03960073, NCT03586414). Regarding the cardioprotective function of MitoQ investigated in animal models and the positive clinical results of protecting vascular function against endothelial injury, MitoQ remains a promising avenue to be investigated in further studies.

Visomitin (SkQ1) is another TPP^+^-binding compound, which prevents the oxidation of mitochondrial cardiolipin, and it is a “rechargeable” antioxidant, which can be reduced by respiratory chain complexes (Skulachev, 2007). Among all the organelles, Skulachev et al. have only observed the exclusive fluorescence of SkQR1 (one of the derivatives of SkQ1) in mitochondria *in vitro*, indicating the high targeted ability of SkQ1 towards mitochondria (Skulachev et al., 2009). SkQ1, similar to MitoQ, exhibits high biological activity even in a nano-molar concentration. However, SkQ1 has even more remarkable anti-oxidation with lower pro-antioxidant activity and wider ranges of working antioxidant activity than MitoQ (Antonenko et al., 2008), which is the reason why SkQ1 is a promising drug target in the treatment of mitochondrial dysfunction. Manskikh et al. showed that SkQ1 alleviated fibrotic formation, rescued the cardiac function, and decreased the incidence of senescence-associated cardiomyopathy in the aged murine heart (Manskikh et al., 2015). Furthermore, a low amount of SkQ1 (0.02 nmol/kg) showed obvious regulation of cardiac rhythm after MI in mice, while relatively high concentrations of SkQ1 (125–250 nmol/kg) helped decrease the infarct area in an I/R model *ex vivo* (Bakeeva et al., 2008). Although there have been no SkQ1-related clinical trials in CVDs, scientists found its benefits in the context of eye diseases. Petrov et al. reported the success of phase II clinical study on SkQ1, showing that it improved the dry eye symptom, which is also consistent with the results in pre-clinical studies (Brzheskiy et al., 2015; Zernii et al., 2017).

Triphenylphosphonium chloride (mitoTEMPO) has been well-studied as a beneficial mitochondrial target in cardiac animal models. As an antioxidant, mitoTEMPO exhibits positive effects on the hearts of aged mice (Olgar et al., 2018). Dey et al. found that mitoTEMPO normalized ROS levels in all cell types and suppressed HF-induced cardiac remodeling in guinea pigs (Dey et al., 2018). In a study by Fang et al., mitoTEMPO rescued iron-dependent CMs death in doxorubicin (DOX)-treated and I/R-induced cardiomyopathy in a murine model (Fang et al., 2019). Besides, mitoTEMPO also has effects against overloaded pressure in a transverse aortic constriction (TAC) model by protecting the integrity of the respiratory chain (Hoshino et al., 2014).

Penetrating peptides, also called Szeto–Schiller peptides, have antioxidant properties in CVDs (Christodoulou et al., 2004). Penetrating peptides are small, transcellular peptides and can be uptake by mitochondria. The underlying mechanism of their antioxidant function may be attributed to tyrosine or dimethyltyrosine residue (Szeto, 2006). One of the representative penetrating peptides is SS-31 (also called elamipretide), which can be accumulated 1,000- to 5000-fold in the IMM and combined with cardiolipin to protect against the damage from ROS (von Hardenberg and Maack, 2017; Szeto, 2014). A previous study has shown the protective function of SS-31 against I/R in a mouse model (Cai et al., 2018). Recently, Whitson et al. observed the reversion of systolic cardiac function in aged mice after treatment with SS-31 for 8 weeks (Chiao et al., 2020). Further research has shown that the treatment with SS-31 together with nicotinamide mononucleotide (NMN, one of the nucleotide precursors of NAD^+^) increases the level of NAD(H) and improves cardiac function in aged mice (Whitson et al., 2020). Furthermore, SS-31 resists mitochondrial dysfunction by blocking the ROS-induced MPTP opening (Zhang et al., 2020). Pre-/clinical studies have also provided prospective outcomes, where SS-31 has been shown to exhibit positive signs of improving cardiac function after acute myocardial infarction (AMI) and angiotensin II infusion (Dai et al., 2011; Dai et al., 2013). Recent phase II clinical trials have focused on the use of SS-31 in the treatment of HF with reduced ejection fraction (HFrEF) or preserved ejection fraction (HFpEF) (Daubert et al., 2017; Butler et al., 2020). In these studies, SS-31 has been proven to have good safety and tolerability in humans. Unfortunately, in the PROGRESS phase II study, no improvement in cardiac function was observed in patients with HFrEF, although the number of enrolled patients was limited. Thus, the function of SS-31 needs to be proven through more clinical studies.

Whether acetyl-L-carnitine confers any cardiac protection remains controversial. In 1999, a double-blind randomized clinical study in 60 patients after AMI showed that L-carnitine had no significant effect in improving left ventricular function. Another study presented similar results after analyzing 4,000 patients (Tarantini et al., 2006); however, in that study, L-carnitine was reported to reduce acute mortality after AMI. In contrast, evidence of cardioprotection of acetyl-L-carnitine was investigated in 2002 by Heger et al., who demonstrated that oral administration of acetyl-L-carnitine promotes the process of β-oxidation and prevents aging-related oxidative stress in older murine hearts (Hagen et al., 2002). Further studies have reported that L-carnitine promotes the activation of SODs and AMPK proteins synthesis (Vacante et al., 2018), thereby protecting from mitochondrial dysfunction. Although acetyl-L-carnitine seems to have no positive influence on cardiac function according to the clinical studies, its role in enhancing cardiac mitochondrial function and protecting against heart aging cannot be estimated. This may represent an interesting target to treat mitochondrial dysfunction in the future.

### 3.2 Targeting Mitochondrial Homeostasis

#### 3.2.1 Targeting Mitochondrial Biogenesis

A previous study has found that widely used angiotensin-converting-enzyme (ACE) inhibitors and angiotensin receptor-II blockers (ARB), which are used to treat hypertension and HF in the clinic, are also beneficial for mitochondrial functions (Muscari et al., 1998). A similar agent can also be found in β-blockers. Carvedilol is a non-selective β-blocker, which has similar properties to α-blockers. Carvedilol can increase cardiac mitochondrial biogenesis *in vivo* (Williams, 1999). Carvedilol improves the function of respiratory chain complexes and regulates ATP balance against cardiac damage in a rabbit model (Sanbe et al., 1995; Gvozdjáková et al., 1999). Notably, carvedilol is a prominent antioxidant compared to other β-blockers, such as atenolol and propranolol, which are not superior to carvedilol in terms of oxidation resistance. Quinn et al. investigated that propranolol inhibits NADH oxidase activity is associated with flavoprotein, whereas, atenolol is only observed a tiny inhibition of a relatively high concentration compared to propranolol in rat hearts. Other studies have demonstrated that atenolol failed to protect hearts under I/R injury by preserving cardiac mitochondria (Lu et al., 1990; Vandeplassche et al., 1991). Although propranolol presents better anti-oxidation than atenolol, propranolol does not affect Δψm and its protection against oxidase activity is negligible (Oliveira et al., 2004). Propranolol does not influence MPTP, and the potential mechanisms of protection come from its hemodynamic effect (Carreira et al., 2006). In contrast to propranolol, described above, carvedilol inhibits heart MPTP at a high-conductance level, and thus prevents swelling of mitochondria and efflux of Ca^2+^ (Oliveira et al., 2003). Oliveira et al. found that the anti-fibrotic function of carvedilol relies on the inhibition of MPTP and calcium overloading (Cheema et al., 2011). Together with its properties of unselected-β and α1-receptors, carvedilol has advantages in CVDs compared to other β-blockers. The underlying mechanisms of carvedilol-induced anti-oxidation are controversial. Yue et al. imply that oxidation resistance of carvedilol is mainly due to its carbazole moiety, and found a direct free radical scavenging pathway located in carbazole of carvedilol. On the opposite, Tadolini et al. demonstrated that the mechanism by which carvedilol scavenges ROS is catalyzed by Fe^3+^ (Tadolini and Franconi, 1998).

Sodium-dependent glucose transporter 2 (SGLT2) inhibitors decrease renal reabsorption of glucose (Heuvel et al., 2002), thereby increasing glucose excretion in the urine. Many clinical and real-world studies have investigated the advantages of a SGLT2 inhibitor in treating diabetic-induced cardiac diseases. In the EMPA-REG OUTCOME study, SGLT2 inhibitor administration exhibited benefits in reducing cardiovascular mortality, hospitalization due to HF, and nonfatal MI in patients with CVDs and diabetes (Zinman et al., 2015). According to experimental studies, SGLT2 inhibitors such as empagliflozin have a positive effect on mitochondrial biogenesis by activating SIRT1 and PGC-1α in a rat model (Maejima, 2019). Besides, inhibiting of SGLT2 rescues the expression level of Mfn2 and Opa1 in the murine heart (Takagi et al., 2018). Similarly, inhibition of Fis1 was observed following in treatment of SGLT2 inhibitor, which avoided ROS production through excessive mitochondrial fission, thereby reducing the infarct area in diabetic hearts *in vivo* (Mizuno et al., 2018). Besides, the SGLT2 inhibitor was also shown to maintain mitochondrial intracellular Ca^+^ homeostasis to improve the antioxidant ability of mitochondria and cardiac functions in a rat model (Olgar et al., 2020).

Another antioxidant found in grapes, resveratrol, has been investigated as an activator of SIRT1. According to Ma et al., resveratrol reversed the mitochondrial biogenesis in diabetic cardiomyopathy (DCM) mice model induced by deletion of SIRT1; the underlying mechanisms of the effect included resveratrol-induced activation of PGC-1α, and further activation of NRF-1, NRF-2, and TFAM (Ma et al., 2017; Kalliora et al., 2019). In addition, resveratrol has a similar function to SIRT3, which is the major regulator of acetylation and deacetylation in the heart (Bagul et al., 2018; Annesley and Fisher, 2019).

#### 3.2.2 Targeting Mitochondrial Dynamics

Mitochondrial division inhibitor 1 (Mdivi1) was first shown to inhibit DRP1 and effect on suppressing excessive mitochondrial fission by decreasing the activity of GTPase Drp1 enzymatic activity and improving mitochondrial fusion (Tanaka and Youle, 2008). However, this conclusion then was challenged by Bordt et al. who found no anti-fission impact of Mdivi on primary neuron cells, but instead, demonstrated its ability to inhibit complex I and reverse electron transfer-mediated ROS (Bordt et al., 2017). Considering the cardioprotective effect of inhibition of complex I in I/R injury (e.g., inhibition of complex I through mitoSNO protects the heart against I/R *in vivo* (Chouchani et al., 2013)), the mechanism of inhibiting either Drp1 or complex I could both contribute to the cardioprotective effect of Mdivi1 in I/R injury. Smith et al.‘s review summarized most of the *in vitro* results of studies on Mdivi1 by 2017. The majority of the results provided positive evidence of Mdivi1-regulated DRP1 fission. They also recommended using live imaging of fission and fusion rates to evaluate mitochondrial dynamics and suggested that further studies should be conducted under strict/suitable control of variables (such as the deletion of DRP1 *in vitro* and the control of the activity of complex I). In CVDs, Mdivi1 contributes to the alleviation of mitochondrial damage through suppressing excessive mitochondrial fission, apoptosis, and enhancing fusion, thereby reducing I/R damage and improving cardiac function (Rosdah et al., 2016). Ishikita et al. proved that the nanoparticle packaged-Mdivi1 decreases the infarct area of the murine heart after I/R damage; the underlying mechanism is that Mdivi1 inhibits DRP1 and DRP1-induced MOMP (Ishikita et al., 2016). The same positive effect of Mdivi1 on the improvement of cardiac function has also been shown in rat models (Ong et al., 2010). However, a clinically relevant pilot study indicated that Mdivi1 had no significant effect on either decreasing infarct area or improving cardiac function in close chest surgery in large rodents (e.g., pig model) (Ong et al., 2019). Mdivi1 also presents its effect on anti-proliferation and cytotoxicity, especially for hyper-proliferative cells. Although evidence *in vivo* showed its cardioprotective function in the heart, we should not ignore that most of these studies involved short-term administration of Mdivi1 (less than 2 h), which is much shorter than studies of cancer cells (ranges from 16 h to 4 days). The duration of treatment may be essential for Mdivi1 to exhibit its cytoprotective and cytotoxic effects (Rosdah et al., 2016). Taken together, the functions of Mdivi1 and its side effects remain to be further investigated.

Dynasore is a small molecule that inhibits the GTPase activity of dynamin and dynamin-related proteins (e.g., DRP1). Gao et al. reported that dynasore is beneficial against I/R injury in the murine heart by inhibiting excessive mitochondrial fission and mitophagy (Gao et al., 2013). It is worthy to notice that the potential pathways of dynasore are beyond GTPase activity. In the regulation of cellular cholesterol, Park et al. demonstrated that dynasore inhibits fluid-phase endocytosis in triple knock out (TKO) of dynamin1, dynamin2, and dynamin3 fibroblasts (Park et al., 2013). Treatment with dynasore presents unique regulation of micropinocytosis, which has not been seen in TKO fibroblasts (Nichols, 2003), suggesting that micropinocytosis is a dynasore-independent process. The function of dynasore on dynamin-like proteins or non-GTPase pathways should be further investigated in the heart.

Yue et al. provided the first evidence in mammals that non-proteolytic ubiquitylation of Mfn1/2 promotes mitochondrial fusion by inhibiting USP30 (a mitochondria-localized deubiquitinase); they also described a small natural molecule, diterpenoid derivative 15-oxospiramilactone (S3), which can promote mitochondrial fusion *via* inhibition of USP30 in Hela’s cells *in vitro* (Yue et al., 2014). However, the role of S3 in cardiac mitochondria remains to be explored.

#### 3.2.3 Targeting Mitophagy

NAD^+^ is a small molecule that plays an essential role in cellular metabolism and DNA repair (Cantó et al., 2015; Yaku et al., 2018). Recently, NAD^+^ has been shown to act as a coenzyme of sirtuins, which participate in multiple pathways of mitophagy (Palmeira et al., 2019). Nicotinamide riboside (NR) is one precursor of NAD^+^ and has been shown to improve cardiac function in a mouse model (Diguet et al., 2018; Liu et al., 2022). Yamaguchi et al. reported that oral NR is effective in increasing NAD^+^ and its intermediate nicotinic acid adenine dinucleotide (NAAD) safely and well-tolerated in the middle-aged and elderly (Yamaguchi and Yoshino, 2016). Several studies proved the safety of NR administration, which supports Yamaguchi’s finding (Mukherjee et al., 2017; Dollerup et al., 2018; Elhassan et al., 2019). Marten et al. investigated the cardioprotective function of NR in reducing systolic blood pressure (SBP) and aortic stiffness (Martens et al., 2018). Further studies are valuable to investigate the effects of NR on CVDs and additional precursors of NAD^+^.

Glucagon-like peptide-1 receptor agonists (GLP-1 RAs) are a standard treatment for type II diabetes due to their hypoglycemic effect and favorable safety in the clinic (Association, 2020). Recent studies have shown beneficial effects of GLP-1 RAs on CVDs through both direct effects (improve vascular function) and indirect effects (control risk factors of CVDs) (Berndt et al., 2021). Liraglutide, a GLP-1 RAs, is an activator of protective mitophagy in rat hearts and injected liraglutide protects against ischemic damage and reduces the fibrotic formation and cardiac remodeling after MI *in vivo* through the SIRT1/Parkin/mitophagy pathway (Qiao et al., 2018).

In addition to the drugs with newly found cardioprotective functions and potential targets supported by clinical trials mentioned above, there are still some natural compounds found in plants or food that have been investigated as potential mitophagy regulators for protecting the heart. Urolithin A is a hydrolyzed metabolite of pomegranate, which is generated by enteric microbiota and represents a potential target of mitochondrial dysfunction in the cardiovascular system through promoting mitophagy (Oh et al., 2020). Urolithin A is the activator of PINK1/Parkin-dependent mitophagy and directly increases the expression of LC3, which is related to the formation of the autophagosome (Palikaras et al., 2018). *In vivo* models have shown the cardiac protection of Urolithin A in DCM through cardiac performance (Savi et al., 2017). Moreover, Tang et al. showed that Urolithin A alleviated I/R injury *via* the PI3K/Akt pathway. According to the research reported by Juan A et al., Urolithin A showed significant anti-atherosclerotic and antiangiogenic functions against CVDs by inhibiting the migration of ECs and suppressing the expression of chemokine (C–C motif) ligand 2 (CCL2) and interleukin-8 (IL-8) (Giménez-Bastida et al., 2012). As Urolithin A is derived from a natural compound and can be accumulated in the myocardium, it may represent a target agent for clinical treatment in the future (Savi et al., 2017).

Recent studies have demonstrated that another natural compound, spermidine, shows cardiac protection *via* enhancing mitochondrial mitophagy. Spermidine can be extracted from food such as broccoli, soybeans, and rice bran (Larqué et al., 2007). Eisenberg et al. showed that food intake of spermidine decreased SBP and delayed the occurrence of HF in Dahl salt-sensitive rats on a high-salt diet (Eisenberg et al., 2016). Oral administration of spermidine in aging mice led to a low level of interleukin-6 (IL-6) and alleviated AS by regulating mitophagy (Tyrrell et al., 2020). Eisenberg et al. found that spermidine was preferentially taken up and accumulated in murine CMs, indicating that spermidine may be a potential target for mitochondrial dysfunction (Nilsson and Persson, 2019). Wang et al. also reported that the function of spermidine in stimulating mitochondrial biogenesis was mediated by increasing SIRT1 and PGC-1α in aged hearts *in vivo* (Wang et al., 2020a). Spermidine prevents mitochondrial dysfunction in CVDs by multiple mechanisms, which makes it a potential substance against mitochondrial dysfunction in clinical studies.

Acacetin is a natural flavone that can be found in snow lotus and other plants. Recent studies have demonstrated its anti-arrhythmic function in different cardiac models *in vitro* and *in vivo* (Chang et al., 2017; Di Diego et al., 2020; Hong et al., 2021). Hong et al. showed that acacetin protected against cardiac senescence by stimulating the PINK1/Parkin pathway and increasing LC3II-critical pathways of mitophagy. Besides, acacetin can also activate SIRT1 and AMPK to revert mitochondrial dysfunction. Although there is not much research on acacetin in CVDs, it can also be a promising target for the treatment of MI (Hong et al., 2021).

Although compounds from food and traditional medicine show impressive cardioprotective results in pre-clinical studies, they are still far away from clinical trials. Firstly, intake from daily food is as high as the effective dosage for treatment and the safety and tolerability of high-dose preparations remain unknown. Secondly, the route of administration and drug delivery system plays essential roles in the pharmacokinetics response, and require further investigation in future studies.

### 3.3 Targeting the AMPK Pathway

AMPK is an exclusive kinase of eukaryotes and is activated by a low concentration of intracellular ATP in various organs (Hardie et al., 2012). AMPK regulates the balance of energy production/consumption by promoting ATP synthesis or restricting ATP depletion (Guigas and Viollet, 2016). AMPK is an essential kinase involved in the regulation of mitochondrial homeostasis, such as mitochondrial biogenesis (PGC-1α) (Guarente, 2007), mitochondrial dynamics (DRP1, MFF), and mitophagy (ULK1, PINK1–PARKIN pathway) (Kim et al., 2011; Scarpulla, 2011; Ducommun et al., 2015; Toyama et al., 2016; Wang et al., 2018). Due to the huge ATP demand, AMPK has a vital role in the prevention/treatment of CVDs. Hence, AMPK is a promising mitochondrial targeting molecule in the cardiovascular system.

Metformin, the most representative medication for type 2 diabetes, has been shown to have beneficial effects on CVDs (El Messaoudi et al., 2013). Various clinical studies have shown the cardioprotective effects of metformin related to diabetes-related HF, I/R, and mortality of AMI (discussed by Aguilar et al.) (Aguilar et al., 2011). Metformin is known as an inhibitor of complex I and restricts infarct area after I/R *in vivo* (Mohsin et al., 2019). In mechanistic studies, metformin has also been proved to be an activator of AMPK. According to Messaoudi et al., metformin can improve cardiac function in diabetic CVDs, which can be attributed not only to the antidiabetic treatment but also to the promotion of cardiac mitochondrial functions *via* AMPK-targeted mitochondrial protection (Whittington et al., 2013). Moheimani et al. showed the protective effects of metformin in I/R rat hearts *ex vivo*, with a significant decrease in the infarct area by modulating phosphorylation of AMPK (Moheimani et al., 2021). According to the review by Varjabedian et al., metformin is a safe clinical drug, with the most serious side effect being acidosis. However, based on the results of numerous clinical studies, the incidence of acidosis is rare and is not only related to the usage of metformin but also the complex conditions in patients with CVDs (Varjabedian et al., 2018). Taking all into consideration, metformin is a promising target against mitochondrial dysfunction in CVDs.

Melatonin is a regulator of circadian rhythms and has shown cardioprotective function in recent studies. Yu et al. showed that melatonin protected from I/R injury in DCM hearts *in vivo* by decreasing mitochondrial fission and enhancing mitochondrial biogenesis and mitophagy *via* SIRT6 and AMPK signaling (Yu et al., 2021). Melatonin alleviates the cardiotoxicity of DOX and protects against ROS and cell death in C57BL/6 mice by activating AMPK/PGC1α (Liu et al., 2018a).

### 3.4 Inhibitors of the MPTP Opening

Cyclosporine A (CsA), a classical immunosuppressant, is an effective inhibitor of MPTP opening and has shown promising results in pre-clinical studies (Griffiths and Halestrap, 1993). Cardioprotection of CsA is dose- and time-dependent. According to these studies, a dosage of 2.5 mg/kg CsA induces cardioprotective function in several (but not all) rodents (Huang et al., 2014). Treatment with CsA is only useful in the first 10–15 min of reperfusion *in vivo* because the MPTP opens transiently during reperfusion (Zalewski et al., 2015). In an early clinical study, Piot et al. reported that CsA is beneficial in ST-segment elevation myocardial infarction (STEMI) patients and decreases the infarct area in a small number of patients (Piot et al., 2008). However, in a phase II CYCLE clinical study, there were no benefits in long-term outcomes for patients with STEMI who underwent percutaneous coronary intervention (PCI) with CsA treatment. Moreover, results from the Phase III CIRCUS clinical study were also disappointing, showing no ability of CsA to decrease either the infarct area or the incidence of adverse events in the long term (Cung et al., 2015; Mewton et al., 2015). The relative homogeneity of the rodent and a repeatable diseased model cannot fully intimate the complicated homeostasis observed in humans, which may explain the failed transition of CsA from bench to bedside. Further clinical studies on CsA should be more precise and require a higher number of participants. CsA has unspecific functions not only targeting MPTP but also calcineurin. Cereghetti et al. believed that the cardioprotection of CsA is due to the regulation of calcineurin but not MPTP (Cereghetti et al., 2008). It also makes sense to explore more specific inhibitors of MPTP opening. Sanglifehrin-A (SFA) is an immunosuppressant, which shows more potent in inhibiting the MPTP opening than CsA. Additionally, SFA has no inhibitory effect on calcineurin (Clarke et al., 2002). Hausenloy et al., found that infusion of SFA during the initial period of reperfusion decreases the infarct area in rat heart and also increases the threshold of ROS for MPTP opening (Hausenloy et al., 2003). Further clinical studies of SFA on CVDs should be conducted.

### 3.5 Targeting miRNA/lncRNA

The number of studies concentrated on noncoding RNAs (ncRNAs) as important regulators in CVDs has greatly increased in recent decades. Numerous studies have shown the positive effects of ncRNA-targeted treatments in pre-clinical studies (Matsui and Corey, 2017). Although these ncRNAs cannot be transcribed and translated into proteins, they have significant effects on chromosome modification, transcription, and post-transcriptional regulation, and thus alter biological functions (Lekka and Hall, 2018). Here, we discussed microRNAs (miRNAs) and long noncoding RNAs (lncRNAs) as relevant targets for cardiac mitochondrial dysfunction.

Both nuclear microRNA (miRNA) and mitochondrial miRNA (mitoMir) have effects on mitochondrial homeostasis (Geiger and Dalgaard, 2017). Mito/miRNA-regulated mitochondrial functions are implicated in CVDs (Wojciechowska et al., 2017). Overexpression of miR-142 has been shown to inhibit protein kinase Cepsilon (PKCε)-mediated cardioprotection *via* targeting mitochondrial ATP-sensitive K^+^ channel, MPTP, ETC, leading to Δψm dissipation and CMs death (Budas and Mochly-Rosen, 2007; Liu et al., 2018b). Suppression of miR-874 alleviates CM necrosis and decreases the infarct area by regulating caspase-8 *in vitro* and *in vivo* (Wang et al., 2013). MitoMirs contribute to mitochondrial dynamics by regulating different targets. For example, miR-30 regulates p53-activated Drp1 (Li et al., 2010); miR-761 inhibits mitochondrial fission by modulation of MFF (Long et al., 2013); miR-499 regulates Drp1 with or without the p53 pathway, and PGC-1α (van Rooij et al., 2009; Wang et al., 2011; Liu et al., 2016), and miR-484 inhibits Fis1-induced excessive mitochondrial fission and apoptosis of CMs (Wang et al., 2012).

LncRNA cytoplasmic endogenous regulator of oxidative phosphorylation 1 (Cerox1) is the first reported lncRNA involved in regulating OXPHOS. Cerox1 is a cytoplasmic lncRNA and promotes the function of respiratory chain complex I in the regulation of proteins and enzymes. Ulteriorly, mitochondrial protection by Cerox1 has been proven both in human tissues and in mice. According to Sirey et al., Cerox1 is expressed in the heart (Sirey et al., 2019), although no study has investigated the cardiac protection of Cerox1. Cerox1 may be a potential target for cardiac mitochondrial dysfunction, but further investigations are needed. LncRNA urothelial carcinoma-associated 1 (UCA1) is specifically expressed in adult hearts (in normal tissue), shows low levels during early AMI, and increases 3 days after AMI. Hence, UCA-1 is believed to be a next-generation biomarker for the diagnosis and prognosis of AMI (Yan et al., 2016). Another study has shown that UCA-1 suppresses excessive ROS generation and cardiac mitochondrial dysfunction *in vitro* (Chen et al., 2019).

LncRNA metastasis-associated lung adenocarcinoma transcript 1 (MALAT1) prevents CMs from mitochondria-dependent apoptosis induced by isoproterenol (ISO) after AMI. Specifically, MALAT1 enhances ULK1-mediated mitophagy in the mitochondria of CMs (Guo et al., 2019). In contrast, Zhang et al. reported another lncRNA, dachshund homolog 1 (DACH1), which aggravates ROS generation and mitochondrial dysfunction. The underlying mechanism involves the bond between DACH1 and SIRT3, which leads to the degradation of ubiquitination. Inhibition of DACH1 may be a promising target of anti-oxidation and may decelerate the progression of DCM (Zhang et al., 2021). Studies of lncRNA in cardiac mitochondria are still limited compared to those of miRNA. In general, ncRNAs show essential roles in cardiac mitochondrial regulation and CVDs. Research of ncRNAs will support new potential strategies from bench to bedside.

## 4 Summary and Perspective

The heart is very dependent on the normal functioning of mitochondria due to its high energy consumption and ATP-sensitive characteristics. Scientists are becoming increasingly aware of the importance of mitochondrial targets for the treatment of CVDs. The topic is not only about the investigations of new targets and their mechanisms, but also about the delivery systems of these drugs. The targets against mitochondrial dysfunction mainly focus on antioxidants, repair of the respiratory chain, and mitochondrial homeostasis (including mitochondrial biogenesis, mitochondrial dynamics, and mitophagy). Furthermore, scientists have gradually accepted that ncRNAs play important regulatory roles in cardiac mitochondria, and future targeted therapies based on ncRNA may become a hot topic (all the targets mentioned in this review and their evidence are summarized in Table 1). However, there remain some issues related to mitochondria-targeted therapies. Firstly, some of the compounds have less effect on dysfunctional mitochondria than expected. For example, TPP^+^-linked modulators (such as MitoQ, MitoTEMPO, and SkQ1) can combine with the OMM due to their lipophilic characteristics, which rely on the accumulation of cationic molecules *via* Δψm (Jiang et al., 2009). However, Δψm is decreased in dysfunctional mitochondria (Zorova et al., 2018), which may reduce the therapeutic efficiency of these targets. Secondly, other targets are presenting accompanying effects during global treatment (tissue-unspecific treatment), the MPTP inhibitor, CsA, representing a good example. Namely, CsA, has shown cardioprotective function in different species/models (Onishi et al., 2012; Badavi et al., 2014; González Arbeláez et al., 2016; Lozano et al., 2020), however, CsA has a significant immunosuppressive effect at the same time (Frank et al., 1999). To solve the issues of low targeting efficiency and accompanying effects, pharmacologically specific targets such as NIM811, which is also an inhibitor of MPTP but has no immunosuppressive effect compared with CsA (Argaud et al., 2005), warrant further investigation. Another way is to establish a more precise way to transport compounds into the heart. Ikeda et al. synthesized nanoparticle-mediated CsA using poly-lactic/glycolic acid (PLGA) nanoparticles, which target injured organs and have shown promising effects on mouse heart models (Ikeda et al., 2016). Therapies based on nanoparticle-mounted targets (even intervention with small-interference RNA of ncRNA) may become a new direction for the treatment of mitochondrial dysfunction and CVDs. Furthermore, the impact of sterile inflammation and immunity induced by damaged mtDNA in CVDs cannot be ignored. The main solution now is to protect mtDNA against ROS damage. Besides, promising PRRs like NLRPs have been well-studied in animal models as a potential target involving inflammation response in CVDs (Molagoda et al., 2021). Clinical drugs, such as metformin and proprotein convertase subtilisin/kexin type 9 (PCSK9) inhibitor, also have the potential to suppress NLRP3-regulated inflammation in the heart (Wang et al., 2020b; Fei et al., 2020). However, targets towards mtDNA and inflammation response involve a vast amount of inflammatory/immune cytokines and pathways. The major problems at present are finding more efficient co-/interventional targets and specific drug delivery systems.

**TABLE 1:** Summary of pre-/clinical evidence of potential mito-targets in CVDs

Targets	Compounds	Type of Research/Models	CVDs	References
	MitoQ	Mouse, rat	HF, I/R	Kondapalli et al. (2012), Ren et al. (2014), Lazarou et al. (2015), Cho et al. (2019), Paradies et al. (2019)
		Clinical trial	Improved vascular function	Smith et al. (1999)
		Ongoing clinical trials	Cardiac function	NCT03960073 NCT03586414
Anti-ROS	SkQ	Mouse, rat	HF, I/R	Adlam et al. (2005), Dare et al. (2015)
	Mito TEMPO	Mouse, guinea pig	HF, I/R	Shill et al. (2016), Braakhuis et al. (2018), Fink et al. (2021)
	SS-31	Mouse	HF, I/R	Antonenko et al. (2008), Bakeeva et al. (2008), Manskikh et al. (2015)
		Phase II clinical trial	No improvement on HFrEF	Fang et al. (2019)
	Acetyl-L-carnitine	Mouse	Defense of aging-related oxidative stress	
		Clinical trial	No improvement on cardiac function, but decreased short-term mortality after AMI	Hoshino et al. (2014)
Biogenesis	SGLT2 (-)	Mouse, rat	Reduced CM death	Sanbe et al. (1995), Gvozdjáková et al. (1999), Williams (1999)
		Clinical trial	Decreased cardiovascular mortality	Vacante et al. (2018)
	Resveratrol	Mouse	DCM	Lu et al. (1990), Vandeplassche et al. (1991)
Dynamics	Mdivi 1	Mouse, rat	I/R, HF	Tadolini and Franconi (1998), Heuvel et al. (2002)
		Pig	No improvement on CM death and cardiac function	Maejima, (2019)
	Dynasore	Mouse	I/R	Takagi et al. (2018)
Mitophagy	NR	Mouse	HF	Tanaka and Youle (2008), Bordt et al. (2017)
		Clinical trial	Reduced SBP and aortic stiffness	Ong et al. (2019)
	Liraglutide	Rat	I/R	Nichols, (2003)
	Urolithin A	Rat	I/R, AS	Cantó et al. (2015), Palmeira et al. (2019)
	Spermidine	Rat	HF, AS	Yamaguchi and Yoshino (2016), Diguet et al. (2018)
	Acacetin	Rat	I/R, HF	Dollerup et al. (2018), Martens et al. (2018)
AMPK	Metformin	Rat	I/R	Nilsson and Persson (2019), Tyrrell et al. (2020)
		Clinical trials	I/R, HF, decreased mortality of AMI	Eisenberg et al. (2016)
	Melatonin	Mouse, rat	I/R, protection of DOX-induced CM toxicity	Di Diego et al. (2020), Hong et al. (2021)
MPTP	CsA	Rat	I/R	Hardie et al. (2012)
		Clinical trial	Decrease infarct area in STEMI patients	Toyama et al. (2016)
		Phase II/III clinical trial	No improvement of decreased infarct area and mortality	Kim et al. (2011); Ducommun et al. (2015)
	SFA	Rat	I/R	Aguilar et al. (2011)
ncRNAs	miR-142	Rat	Decreased CM death and cardiac hypertrophy	Yu et al. (2021)
	miR-874	Rat	Decreased CM death	Griffiths and Halestrap, (1993)
	miR-761	Rat	Decreased CM death	Jin et al. (2019)
	miR-499	Rat	Decreased infarct area	Piot et al. (2008)
	UCA1	H9C2 cells	I/R	Hausenloy et al. (2003)
	MALAT1	H9C2 cells	Decreased CM apoptosis	Matsui and Corey, (2017)
	DACH1	Mouse	DCM	Lekka and Hall, (2018)

Abbreviations: CVD, cardiovascular disease, ROS, reactive oxygen species, HF, heart failure, HFrEF, heart failure with reserved ejection fraction, CM, cardiomyocyte, DCM, diabetic cardiomyopathy, AS, atherosclerosis, DOX, doxorubicin.

Clinical registered numbers were investigated through https://clinicaltrials.gov/
